# Variants in *LAMC3* Causes Occipital Cortical Malformation

**DOI:** 10.3389/fgene.2021.616761

**Published:** 2021-07-20

**Authors:** Xiaohang Qian, Xiaoying Liu, Zeyu Zhu, Shige Wang, Xiaoxuan Song, Guang Chen, Jingying Wu, Yuwen Cao, Xinghua Luan, Huidong Tang, Li Cao

**Affiliations:** ^1^Department of Neurology and Institute of Neurology, Rui Jin Hospital, Shanghai Jiao Tong University School of Medicine, Shanghai, China; ^2^Department of Neurology, Shanghai Jiao Tong University Affiliated Sixth People’s Hospital, Shanghai, China

**Keywords:** occipital cortical malformation, *Lamc3*, childhood-onset seizures, ECM-receptor interaction, PI3K-Akt signaling pathway

## Abstract

Occipital cortical malformation (OCCM) is a disease caused by malformations of cortical development characterized by polymicrogyria and pachygyria of the occipital lobes and childhood-onset seizures. The recessive or complex heterozygous variants of the *LAMC3* gene are identified as the cause of OCCM. In the present study, we identified novel complex heterozygous variants (c.470G > A and c.4030 + 1G > A) of the *LAMC3* gene in a Chinese female with childhood-onset seizures. Cranial magnetic resonance imaging was normal. Functional experiments confirmed that both variant sites caused premature truncation of the laminin γ3 chain. Bioinformatics analysis predicted 10 genes interacted with *LAMC3* with an interaction score of 0.4 (*P* value = 1.0e–16). The proteins encoded by these genes were mainly located in the basement membrane and extracellular matrix component. Furthermore, the biological processes and molecular functions from gene ontology analysis indicated that laminin γ3 chain and related proteins played an important role in structural support and cellular processes through protein-containing complex binding and signaling receptor binding. KEGG pathway enrichment predicted that the *LAMC3* gene variant was most likely to participate in the occurrence and development of OCCM through extracellular matrix receptor interaction and PI3K-Akt signaling pathway.

## Introduction

Malformations of cortical development (MCD) are a series of disrupted cerebral cortex formation disorders caused by multiple etiologies, including genetic, metabolic, vascular, and other factors (Severino et al., 2020). MCD usually occur in early childhood or early adulthood (Raybaud and Widjaja, 2011; Barkovich et al., 2012). It is characterized by developmental delays, intellectual disability, epilepsy, and even cerebral palsy. Occipital cortical malformation (OCCM; MIM: 614115) is a form of MCD characterized by polymicrogyria and pachygyria limited to the occipital lobe. Besides, the seizures, developmental, and cognitive delays can also be observed in affected individuals (Zambonin et al., 2018). A recent study found that impaired visual function was another clinical feature of OCCM due to compromised cortical architecture (Urgen et al., 2019). In 2011, Barak et al. applied whole-exome capture and sequencing technology to identify the recessive or complex heterozygous variants of the gamma-3 chain isoform of laminin (**LAMC3**) caused OCCM (Barak et al., 2011). Thus far, five unrelated families of OCCM caused by **LAMC3** variants have been reported worldwide (Barak et al., 2011; Afawi et al., 2016; Zambonin et al., 2018). Total six variants of **LAMC3** gene were identified in these unrelated families. The clinical features of patients with OCCM caused by **LAMC3** gene variants includes seizures, developmental delay or degeneration and pachygyria and polymicrogyria.

*LAMC3* gene, cloned by Koch et al. (1999), was found to be located on chromosome 9 at q31-34. The *LAMC3* gene encodes the laminin gamma-3 chain, which is made up of 1575 amino acids (Barak et al., 2011). The laminin γ3 chain is widely distributed in different tissue, including the placenta, spleen, skin, reproductive tract, heart, kidney, and brain (Koch et al., 1999; Barak et al., 2011). However, the distribution of the laminin γ3 chain in different organizations has temporal and spatial characteristics. On the one hand, the laminin γ3 chain is located primarily in the skin’s basement membrane, while it is located primarily on the surface of ciliated epithelial cells in the seminiferous tubes and lung (Koch et al., 1999). On the other hand, the expression level of the laminin γ3 chain in the human cortex peaks from late fetal development to late infancy (Barak et al., 2011). However, the study on biological functions and molecular regulatory mechanisms of the laminin γ3 chain in mammals is very limited. For example, the laminin γ3 chain was shown to participate in retinal angiogenesis by regulating astrocyte migration (Gnanaguru et al., 2013).

Here we identified novel compound heterozygous variants in the *LAMC3* gene in a Chinese OCCM patient. Based on thorough clinical and genetic analysis, we further verified the function of the variants *in vitro*. To further explore the molecular mechanisms involved in *LAMC3*, we applied bioinformatics to analyze and predict the pathogenic mechanism of *LAMC3* gene variant. Finally, we summarized the clinical and genetic characteristics of currently reported patients with OCCM due to the *LAMC3* gene variants.

## Materials and Methods

### Genetic Analysis

The genomic DNA of the proband and her parents were extracted using a standardized phenol/chloroform extraction method. Whole exome sequencing was performed as previously described (Zhu et al., 2019). Sanger sequencings was applied to validate variants of the *LAMC3* gene in the proband and her parents. One thousand unaffected individuals of matched geographic ancestry were used as normal controls.

### Cell Culture

HEK 293T cells were cultured in Dulbecco’s Modified Eagle Medium (DMEM) containing 10% fetal bovine serum (FBS) and 1% penicillin/streptomycin (PS) at 37°C with 5% CO2 and 95% O2. *LAMC3* wild-type (*LAMC3*-WT) or variant (c.470G > A) plasmid, synthesized by Gene Create (Wuhan, China), with spliced GFP labels on the N-terminal. Before transfection, cells were plated at 150,000 cells per well in 6-well culture dishes. After 24 h, HEK 293T cells were transfected with 2.5 μg *LAMC3*-WT or variant (c.470G > A) plasmid DNA through Lipofectamine 3000 transfection reagent (Invitrogen). 36 h later, the cells were used for immunofluorescence and western blotting.

### Western Blotting

The concentration of protein samples was determined using a BCA kit. Protein samples were diluted in 5X SDS-PAGE and boiled at 100°C for 10 min. 30 μg of protein sample was resolved by 8% SDS polyacrylamide gels. After being transferred to polyvinylidene fluoride membrane, the blots were incubated at 4°C for 12 h with the anti-GFP antibody (1:1000, Cell Signal Technology) and α-tubulin primary antibody (1:1000, Cell Signaling Technology). Finally, after incubation with secondary antibodies (1:5000, Beyotime), blots were analyzed by Kodak Digital Science1D software (East-man Kodak Co., New Haven, CT, United States).

### Immunofluorescence

After transfection, PBS containing 4% paraformaldehyde was used to fix the cells for 30 min. Cells were blocked using a blocking solution, containing 0.3% Triton X-100 and 5% bovine serum albumin (BSA) in PBS for 30 min. DAPI (1:10,000, Cell Signal technology) was then applied for 5 min to stain nucleic acid staining. Finally, A Zeiss 710 confocal microscope was used for image collection.

### Minigene Reporter Assay

BDGP: Splice Site Prediction by Neural Network,^1^ NetGene2 Sever^2^ and HSF: Human Splicing Finder Version 3.1^3^ were used to evaluate the effects of variants on splice sites. PCR amplified the LAMC3 exons relevant to 1 novel splicing variant (c.4030 + 1G > A) along with flanking intronic sequences from patients P3672 with gDNA. The PCR products of c.4030 + 1G > A were cloned into the pcDNA3.1 vector and the pcMINI vector. Then the minigenes were transfected into HeLa cells and HEK 293T cells, respectively. The RNAiso Plus kit (Takara, Japan) was used for total RNA extraction, followed by RT-PCR using the PrimeScript RT reagent kit (Takara, Japan) to generate related cDNA. Electrophoresis on 2% agarose gels and Sanger sequencing were used for splicing pattern analysis.

### Bioinformatic Analysis

The signal peptide sequence of *LAMC3* was predicted by SignalP-5.0.^4^ The Search Tool for the Retrieval of Interacting Genes database (STRING) online database^5^ database was applied to predict genes potentially associated with *LAMC3*. Protein-protein interaction network (PPI) network and functional enrichment analysis including the cellular component, biological process, molecular function, and KEGG were exported as tables. Sangerbox^6^ and Cytoscape software were used to construct the visualized PPI network diagram and functional enrichment analysis bubble diagram, respectively.

### Ethics

According to the Declaration of Helsinki principles, this study was approved by the Ethics Committee of the Ruijin Hospital affiliated with Shanghai Jiao Tong University School of Medicine. The patient, her parents, and the healthy controls signed informed consent.

## Results

### Case Report

**FIGURE 1:**
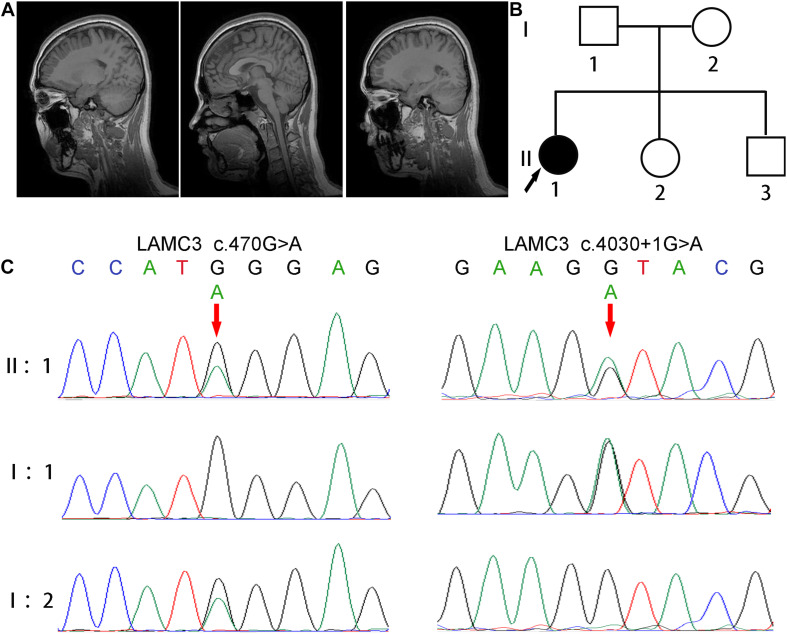
Clinical and genetic characterizations. **(A)** Cranial MRI showed normal brain structure without abnormal signal and lateral ventricular dilation. **(B)** The pedigree of this family shows only the patient had epilepsy, and her parents and two younger siblings were both unaffected. **(C)** Direct polymerase chain reaction sequencing revealed a heterozygous variant c.4030 + 1G > A on exon 24 of the father’s *LAMC3* gene, and a heterozygous variant c.470G > A on exon 2 of the mother’s LAMC3 gene.

A 24 years old female patient, born from non-consanguineous healthy parents with normal development, had epilepsy for 11 years. The patient’s two younger siblings were both unaffected (Figure 1B). According to the parents’ recollection, high fever convulsions for 4–5 min occurred at 9 months and 2 years of age. At the age of 13, the patient first had an absence seizure accompanied by incontinence without obvious inducement. The absence seizure lasted a few minutes and occurred once or twice a month. In addition, generalized tonic-clonic seizures also occurred five times. After each seizure, the patient felt pain on the right side of the head. The patient was treated with valproate at age 20 (500 mg, three times daily). The frequency of absence seizures decreased but still occurred. At the age of 22, the dose of valproate was adjusted to two dose (1000 mg) twice daily. At the same time, the electroencephalogram (EEG) showed 8–9 Hz alpha waves companying with 5–7 Hz theta waves. Cranial magnetic resonance imaging (MRI) was normal (Figure 1A). 3 months later, due to the poor effect of valproate on epilepsy control, the patient was treated with levetiracetam twice a day reducing the dosage of valproate to 500 mg twice daily. After half a year, valproate was discontinued. The most recent follow-up was at age 24. The patient suffered from an absence of seizures twice in the past 2 years. An EEG showed 8–10 Hz alpha waves and 4–7 Hz slow waves.

### Genetic Findings

The patient was revealed to have compound heterozygous variants in the *LAMC3* (NG_029800.1, NM_006059, NP_006050.3) exon 2 c.470G > A/p.Trp157^∗^ and exon 24 c.4030 + 1G > A by whole exome sequencing. Direct polymerase chain reaction and Sanger sequencing were used to verify these variants in the patient and her parents (Figure 1C). A heterozygous variant c.4030 + 1G > A on exon 24 of the father’s *LAMC3* gene, and a heterozygous variant c.470G > A on exon 2 of the mother’s *LAMC3* gene. c.470G > A was found in the Human Mutation Database (HGMD).^7^ c.4030 + 1G > A was not found in the Genome Aggregation Database (gnomAD), 1000 Genome Project, or 1000 healthy controls. The pathogenicity prediction of the two variants was disease-causing for both using Mutation Taster (probability score: 1.0, range: 0–1.0 for both). In addition, c.470G > A was predicted to be damaging in PolyPhen2 and SIFT. Pathogenicity assessment according to the American College of Medical Genetics and Genomics (ACMG), revealed that both the c.470G > A and c.4030 + 1G > A were pathogenic (Richards et al., 2015).

### Functional Analysis

**FIGURE 2:**
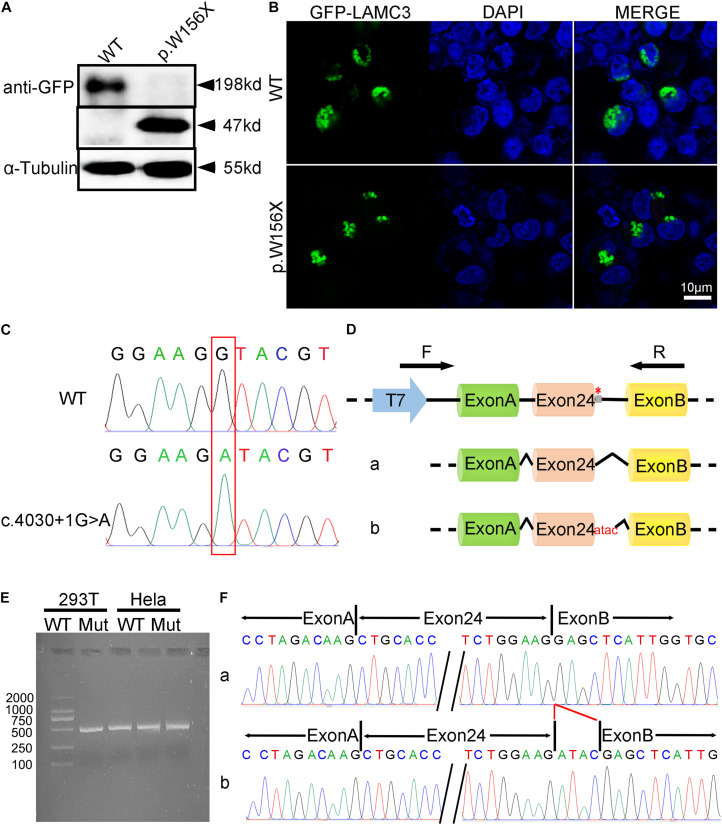
The functional analysis of complex heterozygous variants in *LAMC3* gene. **(A)** The *LAMC3* WT group expressed a normal protein with a molecular weight of 198 kDd and a truncated protein with a molecular weight of 47 kDd in *LAMC3* variant group (c.470G > A). **(B)** The cellular localization of *LAMC3* in WT and variant group was in the cytoplasm without significant morphological difference. **(C)** Sanger sequence of minigene in in WT (above) and variant (below) group (c.4030 + 1G > A). **(D)** The schematic diagrams showed the minigene comprising intron 23, exon 24, and intron 24 of the WT or variant (c.4030 + 1G > A). The variant c.4030 + 1G > A affected the normal splicing of *LAMC3* mRNA, resulting in 4 bp base (ATAC) retention at the left of intron 24, “*” represents variant location. **(E)** Agarose gel electrophoresis of RT-PCR fragments showed a similar length of the product between the WT and variant group. **(F)** The Sanger sequences showed a 4 bp base (ATAC) retention at the left of intron 24 in the variant group.

A truncated protein with a molecular weight of 47 kDa was found in *LAMC3* variant (c.470G > A) transfected cells, while a normal protein with a molecular weight of 198 kDd was found in the WT group (Figure 2A). However, the variant and WT group were both localized in the cytoplasm, and no significant difference was found (Figure 2B). To analyze of the variant c.4030 + 1G > A, three bioinformatics databases (BDGP, NetGene2, and HSF) were adopted to predict the effect of this variant on splicing, and all suggested that the variant would destroy the original Donor and affect the splicing. Then, the minigene comprising intron 23, exon 24, and intron 24 of the WT or variant (c.4030 + 1G > A) were constructed. Both the WT and mutated minigenes were transfected into HEK 293T and HeLa cells to verify this result, respectively (Figures 2C,D). The length of the product detected by RT-PCR in the WT group was consistent with the expected value in both HEK 293T and HeLa cells. However, a similar length of the product was observed in the variant group (Figure 2E). Furthermore, Sanger sequencing of RT-PCR products showed that the variant c.4030 + 1G > A affected the normal splicing of *LAMC3* mRNA, resulting in 4 bp base (ATAC) retention at the left of intron 24 (Figure 2F). The retention of these 4 bp bases further resulted in the production of a truncated protein consisting of 1,367 amino acids.

### Prediction of the Pathogenic Mechanism

**FIGURE 3:**
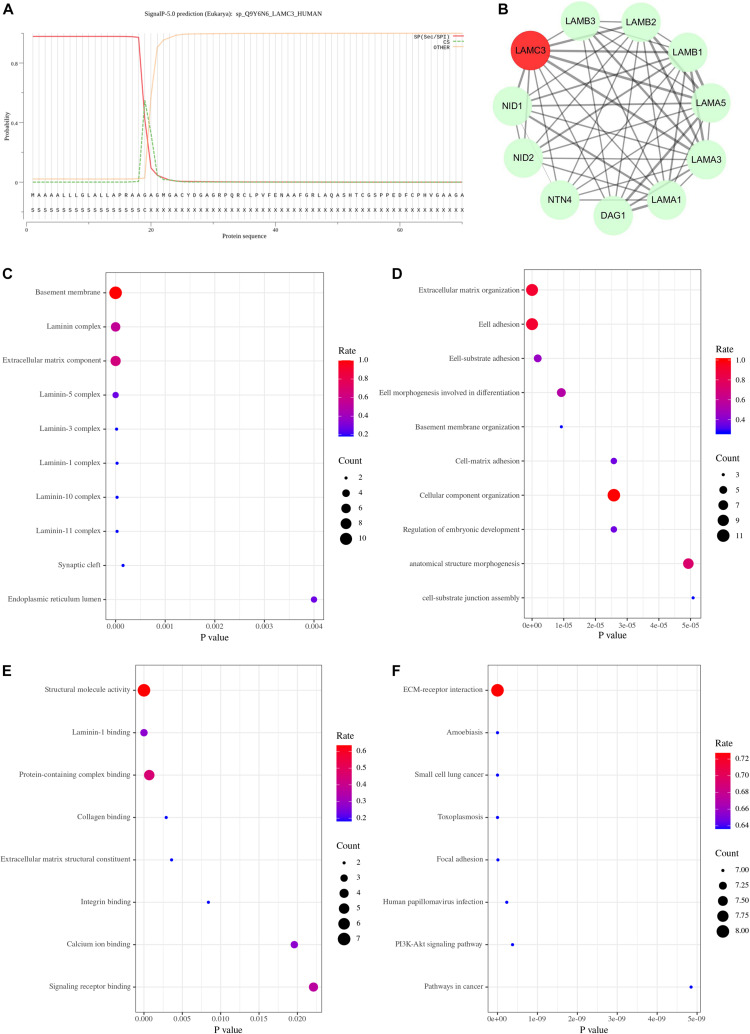
The bioinformatics analysis of LAMC3 gene. **(A)** The first 19 amino acids of the laminin γ3 chain were predicted as a signal peptide. **(B)** The protein-protein interaction network showed 10 genes (*DAG1*, *LAMA1*, *LAMA3*, *LAMA5*, *LAMB1*, *LAMB2*, *LAMB3*, *NID1*, *NID2*, and *NTN4*) that have potential interactions with *LAMC3.* **(C)** The cellular component of gene ontology showed *LAMC3* and its interacting genes were mainly located in the basement membrane, extracellular matrix organization, laminin complex. **(D)** The biological process of gene ontology indicated these genes were mainly associated with extracellular matrix organization, cell adhesion, cellular component organization, and anatomical structure morphogenesis. **(E)** The molecular function of these genes were primarily involved in structural molecule activity, protein-containing complex binding, signaling receptor binding, and laminin-1 binding. **(F)** The KEGG pathway enrichment analysis showed eight potential signal pathways of these genes, including ECM-receptor interaction, amoebiasis, small cell lung cancer, toxoplasmosis, focal adhesion, human papillomavirus infection, PI3K-Akt signaling pathway, and pathways in cancer.

Currently, the mechanism of OCCM caused by *LAMC3* gene variants remained unclear. Therefore, this study intended to explore the pathogenic mechanism through bioinformatic methods. Firstly, online analysis software predicted that the first 19 amino acids of the laminin γ3 chain were the signal peptide sequences of the protein (Figure 3A), which was consistent with reported results (Koch et al., 1999). The STRING online database predicted ten genes (*DAG1*, *LAMA1*, *LAMA3*, *LAMA5*, *LAMB1*, *LAMB2*, *LAMB3*, *NID1*, *NID2*, and *NTN4*) associated with *LAMC3* at the interaction score of 0.4 (*P* value = 1.0e–16) (Figure 3B). The cellular component of gene ontology analysis indicated that *LAMC3* and its interacting proteins were mainly located in the basement membrane, extracellular matrix organization, laminin complex (Figure 3C). In addition, the biological processes of these proteins were involved in extracellular matrix organization, cell adhesion, cellular component organization, and anatomical structure morphogenesis (Figure 3D). Furthermore, the molecular function of gene ontology analysis showed that these proteins were primarily associated with structural molecule activity, protein-containing complex binding, signaling receptor binding, and laminin-1 binding (Figure 3E). Finally, KEGG pathway enrichment showed that ECM-receptor interaction was the most significant pathway and the other pathways contained amoebiasis, small cell lung cancer, toxoplasmosis, focal adhesion, human papillomavirus infection, PI3K-Akt signaling pathway, and pathways in cancer (Figure 3F).

### Literature Review

In 2011, Barak et al. first identified *LAMC3* gene variants as pathogenic variants in three unrelated Turkish families with occipital polymicrogyria and epileptic patients through whole-exome sequencing. Thus far, a total of 12 patients from six unrelated families worldwide (including our patients) have been reported with occipital cortical malformations due to *LAMC3* variants. Genetic and clinical characteristics were summarized in Supplementary Table 1. Firstly, there were seven variants in the *LAMC3* gene that have been identified, including one missense variant, five non-sense variants, and one frameshift variant. The patients in this study carried two variant sites in the *LAMC3* gene: one was c.470G > A, which has been reported by Barak et al. (Barak et al., 2011), and the other was the frameshift variant (c.4030 + 1G > A). This was the first time this frameshift variant has been reported. Seizure was the most common clinical feature of patients with OCCM caused by *LAMC3* gene variants. However, different patients suffered from different types of seizures, including myoclonic-astatic, atypical absence, complex partial, and vision loss. The patient in this study had an absence seizure as the main type of seizure, and had several generalized tonic-clonic seizures. The median age of seizure onset was 10 years old with a range of 2–13 years. In addition, developmental delay or degeneration was another typical clinical feature, including intellectual impairment, speech, fine motor skills delay, and language regression. However, the patient in this study had a normal developmental process with normal cognitive, language, and motor skills. Finally, pachygyria and polymicrogyria were the most representative imaging feature of patients with the *LAMC3* gene variant. The occipital cortex was the most important accumulation site, and the frontal, temporal, and parietal cortex were also reported. Pachygyria and polymicrogyria were not observed in this patient.

## Discussion

OCCM was one kind of MCD characterized by polymicrogyria and pachygyria confined to the occipital lobe and childhood-onset seizures. Studies have found that complex heterozygous or homozygous variants of the LAMC3 gene were responsible for OCCM. This study reported a Chinese female patient with recurrent seizures due to a novel complex heterozygous variant of the *LAMC3* gene. Among them, the non-sense variant (c.470G > A) has been reported by Barak et al. (Barak et al., 2011), while another the frameshift variant (c.4030 + 1G > A) was the first to be identified. Childhood-onset seizures, the most common clinical manifestation of OCCM, were also observed in this patient. However, what was remarkable about this patient was that cranial MRI did not show polymicrogyria and pachygyria in the occipital cortex or other areas. Furthermore, the patient had no developmental delay, including language, motor, and cognitive ability. In terms of treatment, valproate could not control the occurrence of epilepsy in this patient, and levetiracetam achieved better control. This also provides a reference for the treatment of recurrent seizures caused by *LAMC3* variant.

The laminins, major components of basement membranes, are heterotrimeric molecules composed of a combination of different α, β, and γ chains (Durbeej, 2010). Until now, five α chains (α1-α5), three β chains (β1-β3), and three γ chains (γ1-γ3) have been recognized to correspond to different gene products (Tunggal et al., 2000; Durbeej, 2010). The LAMC3 gene (NG_029800.1, NM_006059, NP_006050.3) contains a total of 28 exons, which encodes a laminin gamma-3 chain protein with 1575 amino acids. Thus far, one missense variant, five non-sense variants, and one frameshift variant of the *LAMC3* gene have been identified including the two we found. More importantly, functional experiments demonstrated that both variant sites could cause premature truncation of the laminin γ3 chain. This is consistent with the previously reported effect of variants in the *LAMC3* gene, suggesting the importance of the loss of function caused by premature truncation of laminin γ3 chain in OCCM.

To explore the pathogenic mechanism of the *LAMC3* gene variant, we constructed a protein-protein interaction network with the *LAMC3* gene using bioinformatics. The top ten genes (*DAG1*, *LAMA1*, *LAMA3*, *LAMA5*, *LAMB1*, *LAMB2*, *LAMB3*, *NID1*, *NID2*, and *NTN4*) were predicted to be associated with *LAMC3*. *LAMA1*, *LAMA2*, *LAMC3*, *LAMB1*, and *LAMB2* genes can form different types of laminins through different permutations and combinations with *LAMC3*, such as laminin 213, 323, and 333 trimers (Li et al., 2012). In addition, *LAMC3* was predicted to be related to *NID1* and *NID2*, which was consistent with the previous study. The previous study demonstrated that the laminin γ3 chain was co-located with nidogen-1 and nidogen-2 (Iivanainen et al., 1999; Gersdorff et al., 2005). Furthermore, laminins are the major component of basement membranes, which together with the connective tissue matrix constituted the extracellular matrix (ECM) (Bonnans et al., 2014). The ECM has been shown to play role in structural support, cell differentiation, proliferation, adhesion, migration, and morphogenes (Frantz et al., 2010; Hynes, 2014; Pozzi et al., 2017). Therefore, the laminin γ3 chain, as an integral part of the ECM, plays an important role in these functions. In addition, recent studies on the distribution and function of the laminin γ3 chain in the CNS have found that the laminin γ3 chain was also expressed in the cell body and apical dendrites of the pyramidal neurons on the human temporal occipital region from late fetal development to late infancy (Barak et al., 2011; Zambonin et al., 2018). In the KEGG pathway enrichment analysis, the ECM-receptor interaction was the most significant pathway, which was consistent with the distribution and function of the laminin γ3 chain. Furthermore, the PI3K-AKT signal pathway was predicted as a candidate effector pathway and was activated by growth factors including fibroblast growth factor 2 (FGF2) and insulin-like growth factor 2 (IGF2) to mediate the cell proliferation and growth in the developing brain (Hevner, 2015). However, whether the laminin γ3 chain was involved in the regulation of this pathway remains unclear. It should be noted that studies have shown that the PI3K/AKT pathway was critical in MCD (Iffland and Crino, 2016; Benova and Jacques, 2019). For example, the mutation of the PI3K/AKT pathway was responsible for focal cortical dysplasias (FCDs), hemimegalencephaly (HMEG), and epileptogenic brain malformations (Jansen et al., 2015; Iffland and Crino, 2017). Therefore, the PI3K-AKT signal pathway may be a key pathogenic pathway in OCCM caused induced by the *LAMC3* gene variants. Animal model studies using *LAMC3* gene knockout mice have shown only mild abnormalities. In the nervous system, ectopia of granule cells in the cerebellum, and increased branching of capillaries in the outer retina were observed (Li et al., 2012). In zebrafish embryos, *LAMC3* knockdown resulted in defects in motor neuron guidance (Eve and Smith, 2017). Taken together, these studies suggest that *LAMC3* plays a unique role in the nervous system.

## Conclusion

This work identified novel compound heterozygous variants in the *LAMC3* gene that causes OCCM with recurrent seizures without polymicrogyria and pachygyria. The administration of levetiracetam may be a relatively effective method to control epilepsy associated with *LAMC3* variants. In addition, both variant sites in the *LAMC3* gene could cause premature truncation of the laminin γ3 chain to loss of function. Finally, the extracellular matrix and the ECM-receptor interaction were the main location and signal transduction pathways of the laminin γ3 chain, respectively.

## Data Availability Statement

The raw data supporting the conclusions of this article will be made available by the authors, without undue reservation.

## Ethics Statement

The studies involving human participants were reviewed and approved by the Ethics Committee of Ruijin Hospital affiliated with Shanghai Jiao Tong University School of Medicine. The patients/participants provided their written informed consent to participate in this study. Written informed consent was obtained from the individual(s) for the publication of any potentially identifiable images or data included in this article.

## Author Contributions

LC and HT proposed the idea and designed the study. XQ, XLi, and ZZ completed the experiments. XQ wrote the first draft of the manuscript. XLu provided the clinical patient information. SW, XS, and GC completed part of the manuscript. All authors contributed to manuscript revisions and approved the final version.

## Conflict of Interest

The authors declare that the research was conducted in the absence of any commercial or financial relationships that could be construed as a potential conflict of interest.

## Footnotes

1 http://www.fruitfly.org/seq_tools/splice.html

2 http://www.cbs.dtu.dk/services/NetGene2/

3 http://www.umd.be/HSF3/HSF.shtml

4 http://www.cbs.dtu.dk/services/SignalP/

5 https://www.string-db.org/

6 http://sangerbox.com/

7 http://www.hgmd.cf.ac.uk/ac/index.php as ID, CM113688.
