# Supplementary material for: Variants in LAMC3 Causes Occipital Cortical Malformation
Source: Front Genet. 2021 Jul 20;12:616761. doi: 10.3389/fgene.2021.616761 (PMC8329496; doi:10.3389/fgene.2021.616761)
Supplement: Supplementary file 1 [file Table_1.PDF]

Table 1: Clinical features of patients with LAMC3 mutation.

	Barak et al	Barak et al	Barak et al	Afawi et al.	Zambonin et al	This report
**Mutation**	p.Cys301*	p.Try157*	p.Gly350Arg and p.Gln386*	p.Asp1363Thrfs*54	p.Gln1064*	p.Trp157* and c.4030+1G>A
**Inheritance**	AR	AR	AR	AR	AR	AR
			**Clinical features**			
**Developmental delay**	+	-	NA	+	+	-
**Seizure**	+	+	+	+	+	+
**Age of onset (years)**	2	10	11	NA	3	13
			**Neuroimaging features**			
**Pachygyria**	+	+	+	NA	+	-
**Polymicrogyria**	+	+	+	NA	+	-
**Location**	Parieto-occipital	Occipital	Occipital	Occipital	Frontal, temporal, parietal, occipital	-
**Current treatment**	NA	NA	NA	NA	Levetiracetam and Ethosuximide	Levetiracetam

"+" = positive, "-" = negative, NA=not available, AR: autosomal recessive.
